# ABCB1-Mediated Colchicine Transport and Its Implications in Familial Mediterranean Fever: A Systematic Review

**DOI:** 10.3390/cimb47030210

**Published:** 2025-03-20

**Authors:** Sarah Adriana Scuderi, Alessio Ardizzone, Emanuela Esposito, Anna Paola Capra

**Affiliations:** Department of Chemical, Biological, Pharmaceutical and Environmental Sciences, University of Messina, Viale Ferdinando Stagno D'Alcontres, 31, 98166 Messina, Italy; sascuderi@unime.it (S.A.S.); aleardizzone@unime.it (A.A.); annapaola.capra@unime.it (A.P.C.)

**Keywords:** Familial Mediterranean Fever, *ABCB1*, colchicine, colchicine resistance, drug response

## Abstract

Familial Mediterranean fever (FMF) is an autoinflammatory genetic disorder characterized by recurrent fevers and inflammation of the serous membranes in the abdomen, lungs, and joints. Currently, the standard treatment of FMF includes colchicine, which is an alkaloid, derived from *Colchicum autumnale*. Colchicine’s efficacy in FMF is well-established as it is used both to prevent acute attacks and reduce the risk of long-term complications. However, despite these available treatments, 5–10% of patients exhibit resistance to the drug. It has been demonstrated that polymorphisms in several genes involved in inflammation can influence treatment outcomes and the risk of FMF complications like amyloidosis. Among them, some research focused on polymorphism affecting adenosine triphosphate (ATP)-binding cassette sub-family B member 1 (*ABCB1*) gene encoding for P-glycoprotein. P-glycoprotein is considered a key transporter protein as it regulates the absorption, distribution, and excretion of several drugs, including colchicine. In diseases like FMF, *ABCB1* polymorphisms have been shown to affect the response to colchicine, potentially leading to treatment resistance or altered toxicity. Based on this evidence, this systematic review aims to analyze available evidence on ABCB1-mediated colchicine transport and its clinical implications in FMF, showing how relevant *ABCB1* variants are in response to therapy.

## 1. Introduction

Familial Mediterranean fever (FMF), also called “periodic peritonitis” or “Reimann syndrome”, is an autoinflammatory genetic disorder characterized by recurrent fevers and inflammation of the serous membranes in the abdomen, lungs, and joints, resulting in significant pain [1,2]. The initial episode often occurs in childhood, typically before the age of 20, and it may be accompanied by a rash or headache [1,2].

As the most prevalent of the periodic fever syndromes, FMF predominantly affects individuals of Mediterranean and Middle Eastern descent, which is reflected in its name [1].

FMF is an autosomal recessive disease [1]. The gene responsible for FMF is the *MEFV* gene, which is located on the short arm of chromosome 16 (16 p13.3) [2]. In the last years, approximately 300 different mutations of the *MEFV* gene have been identified and associated to FMF [2].

Currently, the treatment for FMF includes colchicine, an alkaloid obtained from *Colchicum autumnale* [2]. Colchicine reduces the frequency and severity of FMF attacks by decreasing inflammation through neutrophil chemotaxis inhibition [1,3]. Typically prescribed as a daily medication, colchicine can also prevent complications such as amyloidosis, which can arise from prolonged inflammation [2]. However, colchicine can cause several side effects such as diarrhea and vomiting, which are often dose-dependent [2]. Other uncommon side effects are myelosuppression, hepatotoxicity, nephrotoxicity, myopathy, neuropathy, and hypersensitivity reaction [1,3]. Nevertheless, other treatments have been recognized for FMF treatment, such as interleukin-1 (IL-1) inhibitors (e.g., anakinra, canakinumab), which represent the second-line drugs for patients who have colchicine-resistant FMF or who have an intolerance to colchicine [2,4].

Despite these available treatments, it is essential to take genetic polymorphisms into account in the era of personalized medicine. These variations play a vital role in understanding individual responses to therapies, particularly in inflammatory conditions like FMF. In effect, some studies focused on different polymorphisms involved in FMF and treatment response [5,6]. Among them, great attention has been focused on genetic variants affecting adenosine triphosphate (ATP)-binding cassette sub-family B member 1 (*ABCB1*, also known as MDR1 or P-glycoprotein), which is an important membrane transporter that plays a key role in the efflux of various substances out of cells [5,6].

Polymorphisms in the *ABCB1* gene can affect how drugs, including colchicine, are processed in the body, affecting the efficacy and safety of treatment. The clinical effect of these polymorphisms can help to tailor therapies for patients with FMF, ensuring better management of their symptoms and reducing the risk of adverse effects. *ABCB1* polymorphisms may influence also the interaction of colchicine with other medications, further complicating treatment regimens [7,8,9,10].

However, the lack of conclusive data on the influence of *ABCB1* polymorphisms on colchicine response in FMF significantly impacts clinical practice because it limits the ability to tailor treatment and optimize therapeutic outcomes. Without a clear understanding of how genetic variations in *ABCB1* affect the efficacy or toxicity of colchicine, physicians are forced to rely on standard dosage regimens, which may not be suitable for all patients. This systematic review aims to provide a comprehensive summary of the available evidence, paving the way for future research that could clarify the relationship between ABCB1 polymorphisms and colchicine response in order to design customized strategies to improve FMF clinical management. This information would allow the development of more tailored treatment strategies, improving both efficacy and safety for patients with FMF.

The success of pharmacogenetics in other inflammatory diseases, such as rheumatoid arthritis (RA) and inflammatory bowel disease (IBD), where genetic factors like *TPMT* (thiopurine methyltransferase) have already been integrated into clinical practice [11,12], highlights the potential benefits of applying similar personalized approaches in FMF. By leveraging genetic data, clinicians can more accurately predict drug responses and avoid adverse effects, ultimately enhancing patient care.

Thus, in this study, we aimed to explore if variations in the *ABCB1* gene can affect the transport and efficacy of colchicine. In particular, by systematically reviewing existing research, the study seeks to understand the impact of *ABCB1* polymorphism rs1045642 on colchicine treatment outcomes for improving FMF management.

## 2. Methods

### 2.1. Search Strategy

We conducted a comprehensive literature search using the bibliographic databases Web of Science, Embase (via OVID), and PubMed (MEDLINE). To ensure a rigorous search strategy, we adhered to the guidelines outlined in the Preferred Reporting Items for Systematic Review and Meta-Analysis Protocols (PRISMA-P) as previously done [13]. The search was performed by A.A. and S.A.S., focusing exclusively on English-language publications and utilizing the eligibility criteria detailed in Table 1.

Two content experts (A.P.C. and E.E.) oversaw the search strategy development and the research process. The search included articles published from the inception until December 2024. No geographic restrictions were applied. Studies were considered if they focused on ABCB1 transporter linked with colchicine administration in the pathological context of FMF.

The search terms, detailed in Table 2, comprised combinations of keywords related to *ABCB1*, colchicine, and FMF.

### 2.2. Study Selection

We carried out our research using the PubMed (MEDLINE), Embase (OVID), and Web of Science databases to identify relevant records. After the initial search, we removed any duplicate entries. Two review authors (A.A. and S.A.S.) independently screened the titles and abstracts to exclude irrelevant studies. Full-text articles were then thoroughly examined to select those meeting our inclusion criteria. A third reviewer (A.P.C.) was involved in resolving any disagreements between the initial reviewers.

Two authors (A.A. and S.A.S.) performed data extraction from the selected studies. The information collected included study population, geographical location, year of publication, article title, author(s), and associated clinical outcomes.

### 2.3. Assessment of Risk of Bias

Two reviewers (A.A. and S.A.S.) independently assessed the quality of the studies included in this systematic review using the Newcastle–Ottawa Scale (NOS; see Supplementary Table S1). The studies were classified as having a low, moderate, or high risk of bias according to their NOS scores, in line with previous methodologies [14,15,16]. Discrepancies in scoring were resolved through the involvement of a third reviewer (A.P.C.). The following cutoffs were applied: scores below 4 indicated a “high risk of bias”, scores between 4 and 6 represented an “intermediate risk of bias”, and scores above 6 indicated a “low risk of bias”. The following nine criteria of the NOS score were employed: representative of the exposed cohort, selection of external control, ascertainment of exposure, outcome of interest does not present at the start of the study, comparability of cohorts (main factor and additional factor), assessment of outcomes, sufficient follow-up time, and adequacy of follow-up.

Based on these criteria, all the studies were rated as having a “low risk of bias.” The individual assessments for each study are detailed in Supplementary Table S1.

## 3. Results

### 3.1. Findings from Systematic Search

Using our established search strategy, we retrieved a total of 1157 records from PubMed (MEDLINE), Web of Science, and Embase (OVID). After removing duplicate entries, 1005 articles remained. Titles and abstracts were then screened to assess eligibility, which resulted in the exclusion of 997 articles that did not meet the criteria. This left eight articles for full-text review. Studies were excluded during the full-text review phase if they did not meet the inclusion criteria. Four did not fit the required study design, or failed to provide clear data (Supplementary Table S2).

**Figure 1:**
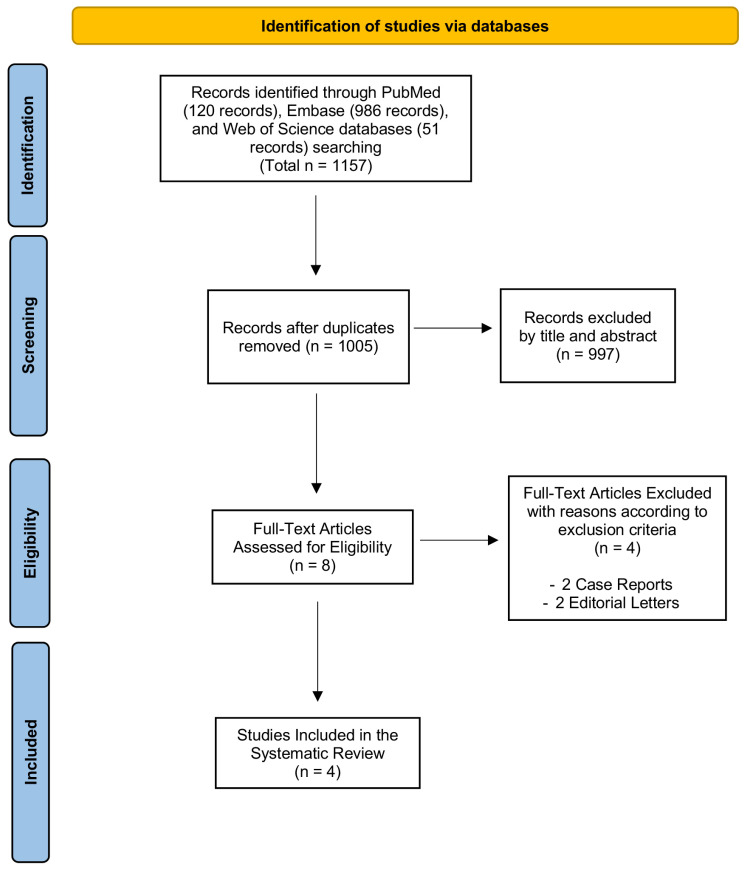
PRISMA flow diagram. The PRISMA flow diagram outlines the entire selection process, starting from the identification stage and concluding with the final inclusion of articles for the systematic review.

Ultimately, as outlined in Figure 1, four studies met the inclusion criteria and were included in this systematic review.

### 3.2. Description of Included Studies in the Systematic Review

This systematic review collected four different studies, all rated as “low risk of bias” following NOS assessment.

In the last years, many single nucleotide polymorphisms of the *ABCB1* gene have been identified. Among these, in particular, the synonymous variant c.3435T>C (p.Ile1145=) in exon 26 (rs1045642) has been reported to be associated with the function and amount of expression of *ABCB1* [6,17]. In this context, Tufan and colleagues [6] evaluated the clinical relevance of this polymorphism in the *ABCB1* gene for colchicine efficacy in 120 FMF patients. Among them, 98 patients were evaluated as responders and 22 as non-responders to colchicine. The distributions of *ABCB1* CC, CT, and TT genotypes were considerably different between responsive and nonresponsive groups [6]. Colchicine resistance was significantly higher in patients harboring the C allele than in patients with the TT genotype. Similarly, the mean colchicine dose to prevent remission was significantly lower in the TT group compared with subjects with the C allele [6].

Ozen et al. [17] studied the association between the C3435T polymorphism and colchicine-resistant FMF patients by using total genomic DNA samples from 52 FMF patients with colchicine unresponsiveness.

The results obtained by Ozen and colleagues showed increased T allele frequency in colchicine non-responder FMF patients, suggesting that C3435T polymorphism was associated with colchicine resistance [17].

Another study by Dogruer and colleagues [18] investigated the impact of *ABCB1* polymorphisms on bioavailability of colchicine in 48 FMF patients showing there was no significant gender difference. Moreover, no significant relationship was found between colchicine doses that would introduce optimal clinical response and *ABCB1* polymorphisms in FMF carrier patients [18].

Later, Uludag and colleagues [5] examined the relationship between *ABCB1* C3435T polymorphism and colchicine response in 50 FMF patients. After the detection of variant C3435T by real-time polymerase chain reaction, patients were divided into three groups: patients who recovered from episodes with standard colchicine treatment and had no attack in the last 1 year defined as complete; patients whose episode number and intensity were decreased with the ongoing standard treatment defined as partial; and patients whose episodes were not decreased despite the standard treatment defined as non-responders [5]. These data suggested that C3435T polymorphism enacts an important role in colchicine response in FMF, showing that good response to colchicine treatment was related to the presence of the C allele, whereas poor response was related to the T allele in FMF-treated patients [5]. The studies cited in this paragraph are summarized in Table 3. The relationship between ABCB1 C3435T polymorphism and colchicine resistance in FMF patients is shown according to PICOTS in Table 4.

## 4. Discussion

In diseases like FMF, *ABCB1* polymorphisms have been shown to affect the response to colchicine, potentially leading to treatment resistance or altered toxicity [19,20].

In this systematic review, data from a total of 270 FMF patients from the Mediterranean area treated with colchicine are collected. No adverse events related to the treatment have been reported.

In the general cohort, all the FMF-treated patients are genotyped for the C3435T variant to evaluate the possible association with drug response. In only three of the four selected studies, we observed a total of 94 FMF patients considered not responding or partially responding to the colchicine treatment. We observed a comparable total number of non-responsive patients between homozygous CC and TT patients (20 vs. 21) and 53 non-responsive patients in the heterozygous group for the C3435T variant in *ABCB1*.

The other 127 FMF patients are considered responsive to colchicine treatment.

In the study by Dogruer et al. [18], we can only report the frequency of this genotype evaluated in a group of FMF patients in treatment with colchicine.

The comprehension of the clinical impact of the variant C3435T in the *ABCB1* gene remains uncertain, but this polymorphism as well as others could help in tailoring the therapy, optimizing efficacy and long-term outcome, especially for help to evaluate in a pre-emptive way the possibility to start a different therapy in case of colchicine resistance.

Canakinumab, which is a monoclonal antibody targeting IL-1β, provides an effective alternative for managing febrile attacks in these resistant cases, even in the absence of certain genetic markers associated with FMF [21]. A recent study suggests that while colchicine is effective for a majority of Japanese FMF patients, a notable subset exhibits resistance. This research underscores the importance of personalized treatment approaches based on individual patient responses and genetic backgrounds [22].

Also, three case studies where FMF patients were either unresponsive or intolerant to conventional treatments showed significant improvement with canakinumab therapy [23].

Another promising therapeutic option for FMF patients is anakinra, an interleukin-1 receptor antagonist, as reported in a randomized controlled trial evaluating the efficacy and safety of anakinra in patients with FMF who do not respond to colchicine treatment [24]. In a retrospective study of 2021, the authors reported a comparison between the two treatments; as stated, both anakinra and canakinumab are effective for FMF management, but canakinumab demonstrates a more favorable safety profile. The potential for serious side effects associated with short- and long-term anakinra usage must be considered in clinical practice [22].

Some limitations should be considered in the current review; firstly, the few included studies. The number of patients is limited, but we have to consider that FMF is an underdiagnosed genetic disorder, prevalent in countries surrounding the Mediterranean Sea, with differences in distribution reported as approximately 1–5 people per 10,000 [25]. Linked to this, we must underline that all the FMF patients in the selected studies are Turkish residents and Mediterranean natives because Turkey is the country with the greatest prevalence of FMF.

Moreover, the collected findings can be subjected to potential bias due to different variables. We included observational studies based on FMF cohorts’ patients that can differ regarding *MEFV* genotype and severity of the disease. Also, age, sex, and other clinical complications, as well as individual pharmacological treatments, can have an impact and confuse the results. The question if this single polymorphism C3435T in the *ABCB1* gene can be associated with a different response to colchicine in FMF patients remains open. More clear evidence will be collected in a larger cohort of FMF patients, and of course, with the use of high-throughput techniques such as Next-Generation Sequencing (NGS), the approach can be more global to better define other pharmacogenetic variants associated with clinical response in colchicine treatment.

## 5. Conclusions

Especially in complex conditions such as inflammatory disorders, every scientific advance can help the life-long clinical management of patients to control their symptoms and prescribe safer and more effective therapeutic options. In this scenario, for FMF patients, new biological drugs targeting interleukin-1 represent a good alternative to the standard colchicine treatment.

A lot of attention is related to the genetic profiles of every FMF patient that can be associated with the severity of the disease and the drug response. In fact, 5–10% of patients exhibit colchicine resistance, and this definition is still controversial also as the underlying mechanism is not fully understood [26]. However, in this clinical setting, it remains mandatory to treat the affecting patients life-long to mitigate their chronic status of inflammation. To date, canakinumab and anakinra represent a significant therapeutic option for managing various inflammatory disorders, particularly those involving dysregulated IL-1 activity, including FMF.

For this reason, it is useful to collect data on the presence of polymorphisms that can guide therapeutic choices in a rapid and personalized manner’ in this case, we consider a synonymous variant C3435T in the *ABCB1* gene associated with an altered expression of a transport protein that regulates the efflux of colchicine from cells.

In the future, more evidence resulting from integrated data about genetics, pharmacology, and clinics would help to tailor the treatments and improve FMF patients’ quality of life.

## Figures and Tables

**Table 1:** Inclusion and exclusion criteria employed for the literature search.

Inclusion Criteria	Exclusion Criteria
Clinical trials, randomized controlled trials, observational studies, case-control studies, cross-sectional studies, cohort studies.	Case reports, editorials, letters, reviews, guidelines, abstracts and paper conferences, systematic reviews and meta-analyses, and ongoing studies. Articles not written in English.

**Table 2:** Combinations of keywords used for the search plan.

FMF	Colchicine	ABCB1
“Familial Mediterranean Fever”, “Familial Mediterranean Fevers”, “FMF”, “FMFs”, “Mediterranean Fever”, “Mediterranean Fevers”, “Familial Auto-inflammatory Syndrome”, “Familial Auto-inflammatory Syndromes”, “Hereditary Mediterranean Fever”, “Hereditary Mediterranean Fevers”, “Mediterranean Fever Syndrome”, “Mediterranean Fever Syndromes”, “familial paroxysmal polyserositis”, “periodic peritonitis”, “recurrent polyserositis”, “benign paroxysmal peritonitis”, “periodic disease”, “periodic fever”, “Reimann periodic disease”, “Reimann syndrome”, “Siegal-Cattan-Mamou disease”, “Wolff periodic disease”,	“Colchicine”, “Colchicines”, “Alkaloid colchicine”, “Alkaloid colchicines”, “Colchicine drug”, “Colchicine drugs”, “Colchicine compound”, “Colchicine compounds”.	“ABCB1”, “MDR1”, “P-glycoprotein”, “P-gp”, “PGP”, “ATP-binding cassette sub-family B member 1”, “P-glycoprotein transporter”, “Multidrug resistance gene 1”, “ABC transporter B1”.

**Table 3:** Overview of the included studies.

First Author and Year of Publication	Design of the Study	FMF Patients Included (n)	Outcomes	Reference
Dogruer et al., 2013	Observational	48	No significant gender difference in the *ABCB1* polymorphism has been revealed.	[18]
Ozen et al., 2011	Observational	52	The mutated T allele frequency was higher than (0.596) the wild C allele (0.404) in the current colchicine unresponsiveness FMF patients.	[17]
Tufan et al., 2007	Observational	120	Cytopenia, liver function abnormalities, and elevated creatine kinase levels were observed in 9, 24, and 13 cases, respectively. All side effects were mild.	[6]
Uludag et al., 2014	Observational	50	Risk of poor colchicine response in subjects with T allele was increased 2.45 times more than in the C allele.	[5]

**Table 4:** Relationship between ABCB1 C3435T polymorphism and colchicine resistance in FMF patients according to PICOTS.

	Dogruer et al., 2013 [18]	Ozen et al., 2011 [17]	Tufan et al., 2007 [6]	Uludag et al., 2014 [5]
P	48 FMF patients (27 female and 21 male), mean age 33.24 ± 12.32	52 FMF patients (23 female and 29 male), ages ranging from 7 to 41 years; nonresponsive (NR).	120 FMF patients (68 Female and 52 Male). Responsive (R): 56 female and 42 male; mean age 31.5 ± 9.6. Nonresponsive (NR): 12 female and 10 Male; mean age 33.3 ± 9.8.	50 FMF patients (data for each participant can be requested, but are not reported in the study). N = 30 responsive (R), N = 20 nonresponsive (NR) or partially responding (PR).
I	Colchicine 0.5 mg in tablets	Colchicine from 1 to 3 mg/daily	Colchicine ≥ 2 mg/daily	Standard colchicine dose
C	No control group	No control group	84 healthy subjects	No control group
O	C3435T polymorphism. C/C patients N = 1, C/T patients N = 26, T/T patients N = 21.	C3435T polymorphism. C/C patients N = 9 NR, C/T patients N = 24 NR, T/T patients N = 19 NR.	C3435T polymorphism. C/C patients N = 31: N = 23 R; N = 8 NR, C/T patients N = 57: N = 44 R; N = 13 NR, T/T patients N = 32: N = 31 R; N = 1 NR.	C3435T polymorphism. C/C patients N = 20: N = 17 R; N = 3 PR. C/T patients N = 27: N = 11 R; N = 16 NR or PR. T/T patients N = 3: N = 2 R; N = 1 PR,
T	Not clearly indicated	Not clearly indicated	1 year of study	Not clearly indicated
S	Observational study	Observational study	Observational study	Observational study

P = population; I = intervention; C = comparator; O = outcome, T = timing; S = study design. R = responsive; PR = partially responding; NR = nonresponsive.

## Supplementary Materials

The following supporting information can be downloaded at: https://www.mdpi.com/article/10.3390/cimb47030210/s1. Ref. [27] are cited in Supplementary Materials file.

## Abbreviations

FMF	familial Mediterranean fever
ABCB1	adenosine triphosphate (ATP)-binding cassette sub-family B member 1
IL-1β	interleukin-1β
