# Supplementary material for: ABCB1-Mediated Colchicine Transport and Its Implications in Familial Mediterranean Fever: A Systematic Review
Source: Curr Issues Mol Biol. 2025 Mar 20;47(3):210. doi: 10.3390/cimb47030210 (PMC11941201; doi:10.3390/cimb47030210)
Supplement: Supplementary file 1 [file cimb-47-00210-s001.zip › Supplementary Table S1.pdf]

RISK OF BIAS Table	SELECTION				COMPARABILITY		OUTCOME			
	Representative of the exposed cohort	Selection of external control	Ascertainment of exposure	Outcome of interest not present at the start of the study	Comparability of Cohorts		Assessment of outcomes	Sufficient follow-up time	Adequacy of followup	
					Main Factor	Additional Factor				
	1	2	3	4	5	6	7	8	9	OVERALL
Dogruer et al., 2013	X		X		X	X	X	X	X	7
Ozen et al. 2011	X		X		X	X	X	X	X	7
Tufan et al. 2007	X	X	X		X	X	X	X	X	8
Uludag et al. 2014	X		X		X	X	X	X	X	7

Supplementary Table S1. Table with Risk of Bias judgements according NOS score
