# Supplementary material for: ABCB1-Mediated Colchicine Transport and Its Implications in Familial Mediterranean Fever: A Systematic Review
Source: Curr Issues Mol Biol. 2025 Mar 20;47(3):210. doi: 10.3390/cimb47030210 (PMC11941201; doi:10.3390/cimb47030210)
Supplement: Supplementary file 1 [file cimb-47-00210-s001.zip › Supplementary Table S2.pdf]

Article citation in NLM format	Exclusion reason
Ben-Chetrit E, Aamar S. About colchicine compliance, resistance and virulence. Clin Exp Rheumatol. 2009 Mar-Apr;27(2 Suppl 53):S1-3. PMID: 19796522.	Editorials were not admitted based on the inclusion criteria of our study
Bezalel Y, Gershoni-Baruch R, Dagan E, Lidar M, Livneh A. The 3435T polymorphism in the ABCB1 gene and colchicine unresponsiveness in familial Mediterranean fever. Clin Exp Rheumatol. 2009 Mar-Apr;27(2 Suppl 53):S103-4. PMID: 19796545.	Letters were not admitted based on the inclusion criteria of our study
Cohen O, Locketz G, Hershko AY, Gorshtein A, Levy Y. Colchicine-clarithromycin-induced rhabdomyolysis in Familial Mediterranean Fever patients under treatment for Helicobacter pylori. Rheumatol Int. 2015 Nov;35(11):1937-41. doi: 10.1007/s00296-015-3325-y. Epub 2015 Jul 26. PMID: 26210999.	Reviews were not admitted based on the inclusion criteria of our study
Toshida M, Konishi Y, Ikenouchi A, Okamoto N, Yoshimura R. Colchicine-Resistant Familial Mediterranean Fever With Depressive State Successfully Treated With Escitalopram. Cureus. 2021 May 20;13(5):e15145. doi: 10.7759/cureus.15145. PMID: 34164245; PMCID: PMC8214656.	Case reports were not admitted based on the inclusion criteria of our study

Supplementary Table S2. The 4 articles not included are reported with the related reason, according to PRISMA guidelines.
